# Hyperactive Neuroendocrine Secretion Causes Size, Feeding, and Metabolic Defects of *C. elegans* Bardet-Biedl Syndrome Mutants

**DOI:** 10.1371/journal.pbio.1001219

**Published:** 2011-12-13

**Authors:** Brian H. Lee, Jason Liu, Daisy Wong, Supriya Srinivasan, Kaveh Ashrafi

**Affiliations:** 1 Department of Physiology and the UCSF Diabetes Center, University of California, San Francisco, San Francisco, California, United States of America; 2 Department of Molecular and Experimental Medicine, The Scripps Research Institute, La Jolla, California, United States of America; Stanford University Medical Center, United States of America

## Abstract

Bardet-Biedl syndrome, BBS, is a rare autosomal recessive disorder with clinical presentations including polydactyly, retinopathy, hyperphagia, obesity, short stature, cognitive impairment, and developmental delays. Disruptions of BBS proteins in a variety of organisms impair cilia formation and function and the multi-organ defects of BBS have been attributed to deficiencies in various cilia-associated signaling pathways. In *C. elegans*, *bbs* genes are expressed exclusively in the sixty ciliated sensory neurons of these animals and *bbs* mutants exhibit sensory defects as well as body size, feeding, and metabolic abnormalities. Here we show that in contrast to many other cilia-defective mutants, *C. elegans bbs* mutants exhibit increased release of dense-core vesicles and organism-wide phenotypes associated with enhanced activities of insulin, neuropeptide, and biogenic amine signaling pathways. We show that the altered body size, feeding, and metabolic abnormalities of *bbs* mutants can be corrected to wild-type levels by abrogating the enhanced secretion of dense-core vesicles without concomitant correction of ciliary defects. These findings expand the role of BBS proteins to the regulation of dense-core-vesicle exocytosis and suggest that some features of Bardet-Biedl Syndrome may be caused by excessive neuroendocrine secretion.

## Author Summary

Bardet-Biedl syndrome, BBS, is a rare human genetic disease caused by mutations in many genes. The BBS phenotype is very complex; it is principally characterized by early-onset obesity, progressive blindness, extra digits on the hands and feet, and renal problems. BBS patients may also suffer from developmental delay, learning disabilities, diabetes, and loss of the sense of smell. This complexity suggests that BBS proteins function in a variety of tissues, causing defects in many organs. A unifying theme for the diverse features of BBS emerged when BBS genes were identified and their protein products were found to function in the cilium, a sensory structure found in many cell types. Since then, the various manifestations of BBS have been attributed to the loss of ciliary function in the corresponding tissues. This notion was also supported by the finding that mutations in several genes required for proper cilia formation and function reproduce some of the features seen in BBS patients. Here, we have further investigated the defects found in *Caenorhabditis elegans* strains carrying mutations in BBS genes (*bbs* mutants). We find that not only do they display sensory deficits associated with loss of ciliary function, but they also exhibit increased release of multiple peptide and biogenic amine hormones contained in dense-core vesicles of ciliated sensory neurons. Importantly, limiting this excessive hormonal release without correcting the ciliary defects of *bbs* mutants was sufficient to restore normal body size, feeding, and metabolism to these mutants. Moreover, we show that although non-*bbs* ciliary mutations can mimic some of the phenotypes of *bbs* mutants, these effects can be attributed to distinct spatial and molecular mechanisms. Our findings indicate that *C. elegans bbs* mutants exhibit features of both ciliary and endocrine defects and suggest that some of the clinical manifestations of human BBS may result from excessive endocrine activity, independently of the loss of ciliary function.

## Introduction

Bardet-Biedl Syndrome is a rare, multigenic, pleiotropic disorder characterized by defects in many tissues including the eyes, kidneys, central nervous system, and reproductive organs [1]. The clinical presentations of BBS include broadly prevalent disease conditions such as obesity and retinopathy. Mutations in at least 14 genes cause BBS [1]–[3]. Of these, seven interact to form a complex termed the BBSome [4]. The mammalian BBSome associates with ciliary membranes and interacts with a guanine nucleotide exchange factor (GEF) for Rab8, a Rab GTPase implicated in cilia formation and function [4]. The BBSome is thought to act as a coat protein for cilia-bound vesicles, promoting the sorting and delivery of signaling molecules to the cilium [5]–[7]. Given that cilia are sensory organelles enriched in signaling molecules, the molecular functions of the BBSome have led to the current paradigm for understanding Bardet-Biedl Syndrome in which defects in cilia formation and signaling have been advanced as a unifying explanation for the wide range of phenotypes seen in BBS patients [3],[8]–[11]. In support of this notion, conditional knockouts in components of intraflagellar transport, IFT, a multi-protein machinery required for building the cilium, have been shown to result in some of the phenotypes present in *bbs*-deficient mice such as obesity and kidney disease [12]–[14].

In *C. elegans*, the seven BBSome components are conserved and are exclusively expressed in the 60 sensory neurons of this animal [8]. Various subsets of these neurons have been implicated in sensation of environmental cues that affect animal growth, metabolism, and feeding behavior [15]–[17]. Disruptions of the *C. elegans* BBSome cause altered rates of IFT, defects in the structural integrity of sensory cilia, and diminished behavioral responses to various sensory cues [8],[9],[18],[19]. Thus, in *C. elegans* as in mammals, loss of BBSome components leads to phenotypes that are also seen in IFT mutants. Consequently, the physiological abnormalities of *C. elegans bbs* mutants have also been attributed to defects in cilia formation and signaling [9],[18].

Here we report that BBSome mutants display a dramatic increase in the secretion of dense-core vesicle cargoes from ciliated sensory neurons, while mutations in IFT components generally result in reduced secretion, indicating that the consequences of *bbs* deficiency cannot be fully explained by impaired intraflagellar transport. We show that the enhanced secretion of *bbs*-deficient animals depends on the evolutionarily conserved Rab27/rabphillin/CAPS exocytosis machinery but not on Rab8, which participates in vesicular transport to the cilium. Importantly, we show that the altered size, feeding, and metabolic phenotypes of *bbs* mutants can be normalized to wild-type levels by abrogating the enhanced secretion of these mutants without simultaneous correction of ciliary defects. We also demonstrate that while certain phenotypes of IFT mutants mimic those caused by *bbs* mutations, distinct neural and molecular mechanisms underlie these phenotypes. These findings expand the role of the BBSome to the regulation of dense-core vesicle secretion and raise the possibility that enhanced neuroendocrine secretions rather than ciliary defects *per se* may be primarily responsible for some of the clinical features of BBS patients.

## Results

### *C. elegans* BBSome Mutants Exhibit Increased Secretion of Insulins and Neuropeptides

To survive and thrive in a dynamic environment, *C. elegans*, as in other animals, must sense changes in environmental conditions and respond through behavioral, physiological, and metabolic adaptations. *C. elegans* accomplishes this in part by secreting a diverse array of neuroendocrine hormones including insulin-like peptides from their various ciliated sensory neurons [20]. We had previously shown that insulin secretion from the ADL pair of ciliated sensory neurons can be assessed by expressing a fluorescently tagged insulin, DAF-28-mCherry, exclusively in this pair of neurons, and measuring the accumulation of the secreted insulins in coelomocytes, scavenger cells that non-specifically endocytose pseudocoelomic fluid [21]. It is thought that peptidergic secretions from neurons such as ADL, which are not directly in contact with the pseudocoelom, gain access to this space through the extracellular matrix and the ancillary pseudocoelom, a fluid fill cavity that bathes many of the neurons in the head of the worm. Eventual passage of secreted molecules from the ancillary pseudocoelom to the pseudocoelom is thought to be mediated by tight junctions. Coelomocytes within the pseudocoelom non-specifically endocytose pseudocoelomic fluids, concentrating them in endocytic vesicles; thus, the steady state level of circulating signaling molecules secreted from ADL can be measured by quantifying the amount of fluorescent insulins within the coelomocytes [21]–[23].

**Figure 1:**
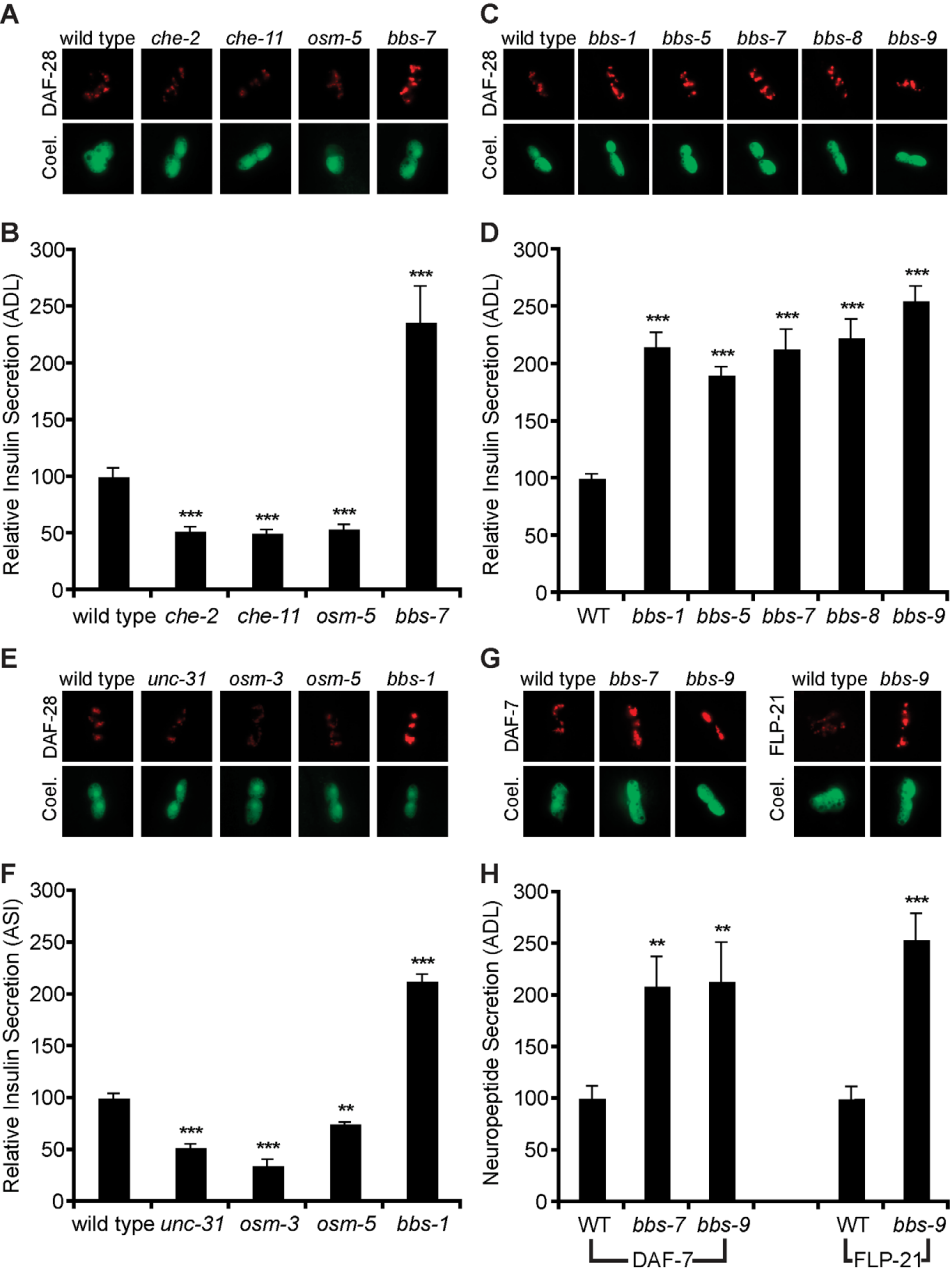
Loss of BBSome increases release of insulins and neuropeptides. (A, C, E, G) Representative images of tagged insulin (DAF-28) or neuropeptide (DAF-7, FLP-21) accumulation in coelomocytes (coel.), and (B, D, F, H) corresponding quantitation with standard error (*** *p*<0.001, ** *p*<0.01 compared to wild type). In each case, indicated insulin or neuropeptides were tagged with mCherry, while *Punc-122::GFP* was used to mark coelomocytes.

To better understand the molecular mechanisms that link environmental cues to secretion of insulin-like peptides, we examined the effects of various defects in cilia formation and function on insulin secretion from the ADL pair of sensory neurons. We found that mutations in intraflagellar transport, IFT, components such as *osm-3*, *osm-5*, *che-2*, or *che-11* [24], which result in defective cilia, cause a ∼50% reduction in insulin secretion (Figure 1A,B,E,F) consistent with the idea that appropriate cilia function is required for detection of food-related cues and subsequent coordination of metabolic and growth pathways through insulin signaling. We were therefore surprised to find that cilia-defective *osm-12* mutants [18], the *C. elegans* homolog of *bbs-7*, exhibit a dramatic 2–3-fold increased insulin accumulation in coelomocytes (Figure 1A–D). Mutations in *bbs-1*, *bbs-5*, *bbs-8*, and *bbs-9*, encoding other components of the BBSome, also caused enhanced accumulation of the insulin reporter in coelomocytes (Figure 1C,D), suggesting that the entire BBSome complex regulates insulin release from ADL sensory neurons.

The increased secretion was not limited to ADL neurons, as *bbs* mutants also exhibited increased secretion when the fluorescent insulin reporter was expressed exclusively in the ASI pair of sensory neurons (Figure 1E,F). Additionally, BBSome mutants exhibited a ∼2–3-fold elevated secretion of DAF-7, a neurally expressed TGF-β ligand and FLP-21, an FRMF-amide neuropeptide expressed under the ADL specific, *srh-220*, promoter (Figure 1G,H), indicating that BBSome mutants have elevated release of various dense-core vesicle cargoes from multiple ciliated sensory neurons.

**Figure 2:**
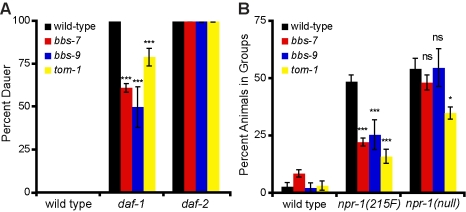
BBSome mutants exhibit increased insulin and neuropeptide signaling. (A) BBSome and *tom-1* mutants partially suppress the dauer arrest of TG-β receptor *daf-1*, but not the insulin receptor, *daf-2* mutants. The percentage of dauers is shown along with standard error of the mean (*** *p*<0.001 compare to *daf-1* animals). (B) BBSome and *tom-1* mutants partially suppress the hypomorphic (215F) allele but not the null allele of the NPY-like receptor *npr-1*. Percentage of animals feeding in social groups is shown with standard error (*** *p*<0.001, * *p*<0.05; *ns*, not significant, comparing double mutants with the corresponding *npr-1* single mutant).

To assess whether the enhanced accumulations of the fluorescent reporters in coelomocytes indicate functional increases in the release of insulins and neuropeptides, we assayed phenotypes associated with hyperactive insulin and neuropeptide signaling. Insulin and TGF-β signaling pathways regulate whether *C. elegans* grow reproductively or enter a hibernating dauer stage [25]. Reductions in either of these parallel pathways initiate dauer entry, while favorable conditions promote dauer exit and reproductive growth. Accordingly, overexpression of either *ins-4* or *daf-28*, encoding distinct insulins, partially suppresses the constitutive dauer formation phenotype of TGF-β mutants [22]. We found that mutations in either *bbs-7* or *bbs-9* partially suppressed the dauer phenotypes of *daf-7* or *daf-1* mutants, encoding the TGF-β ligand and its receptor, respectively, but not that of mutants in *daf-2*, the insulin receptor (Figure 2A and unpublished data). Consistent with the notion that the enhanced secretion of *bbs* mutants mediates dauer suppression, loss of *tom-1*, an inhibitor of the dense-core vesicle release [26], also partially abrogated dauer formation of TGF-β mutants (Figure 2A). By contrast, mutations in IFT components such as *osm-5* and *che-11* failed to alter dauer formation of TGF-β mutants [27]. Therefore, BBSome mutants display enhanced insulin signaling and have the same impact on dauer formation and maintenance as animals with enhanced dense-core vesicle secretion and as such are distinct from other ciliary defective mutants.

To functionally assess neuropeptide signaling in *bbs* mutants, we examined the NPR-1 signaling pathway. FLP-21 is one of two FRMF-amide neuropeptides that activates NPR-1, a neuropeptide-Y-like receptor that regulates *C. elegans* oxygen sensation and social feeding [28]. Overexpression of FLP-21 can partially suppress the hypomorphic *npr-1(215F)* but not the null mutation of *npr-1* as the hypomorph can still bind to FLP-21, albeit with a lower affinity [28]. We found that mutations in either *bbs-7* or *bbs-9* also partially suppress the social feeding phenotype of *npr-1(215F)* but not that of the null mutant, suggesting that BBSome mutants, as our reporter assay indicated (Figure 1G,H), have increased FLP-21 release (Figure 2B). The hypersecreting *tom-1* mutants similarly strongly suppressed the hypomorphic allele of *npr-1* but only weakly suppressed the null allele (Figure 2B). These findings indicated that the enhanced secretion of neuropeptides as assessed by fluorescent reporters indeed correlate to functional increases in neuropeptide signaling in BBSome-deficient animals.

### BBSome Acts in Part Cell Autonomously to Regulate Secretion

**Figure 3:**
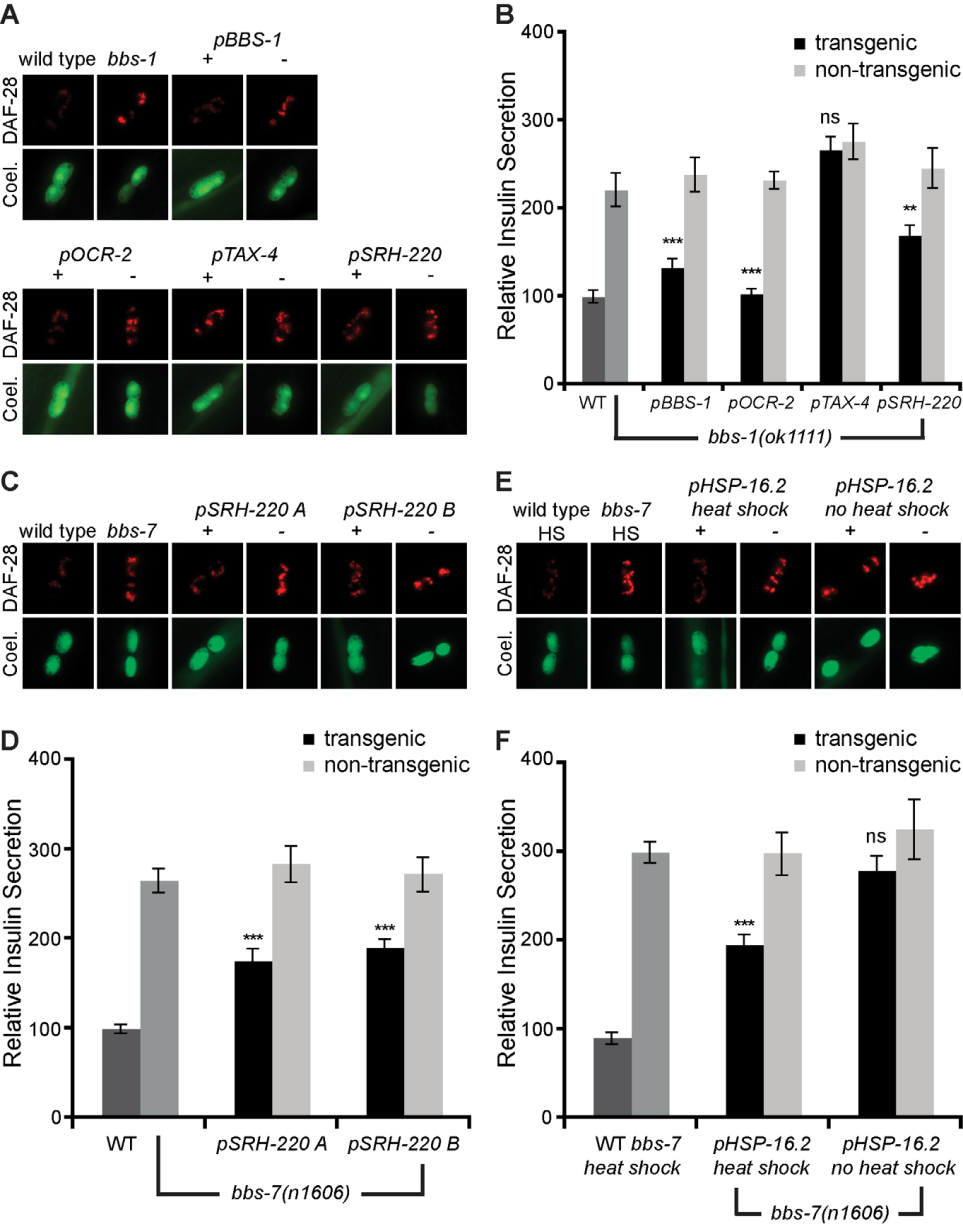
BBSome regulate secretion partially cell autonomously. (A, C, E) Representative images of tagged insulin accumulation in coelomocytes, and (B, D, F) corresponding quantitation with standard error (*** *p*<0.001, ** *p*<0.01; ns, not significant, comparing transgenic to non-transgenic siblings). (A, B) Transgenic (+) and non-transgenic siblings (−) expressing *bbs-1* cDNA under the indicated promoters. Expression patterns of these promoters are listed in Table S1. Transgenic (+) and non-transgenic siblings (−) expressing *bbs-7* cDNA exclusively in ADL using the *srh-220* promoter (C, D) or the heat shock inducible, *hsp-16.2*, promoter (E, F).

To determine if the effects of the BBSome on dense-core vesicle secretion are cell-autonomous, we expressed wild-type *bbs-1* cDNA under the control of various sensory neuron promoters in *bbs-1(ok1111)* mutants. This *bbs-1* transgene was fully functional as judged by its capacity to fully rescue the small body size, feeding, and dye-filling defect of *bbs-1* mutants (Table S1; unpublished data). Expression of *bbs-1* under its own promoter or that of *ocr-2*, which drives expression in ADL in addition to a few other sensory neurons (see Table S1 for expression pattern), abrogated the enhanced secretion of *bbs-1* mutant animals (Figure 3A,B). By contrast, use of the *tax-4* promoter to drive expression in numerous sensory neurons excluding ADL had no effect on the secretion phenotype of *bbs-1* mutants (Figure 3A,B). Expression exclusively in ADL neurons showed partial but highly significant reduction in the enhanced insulin secretion of *bbs-1* mutants, suggesting that the BBSome regulates secretion at least partially cell autonomously (Figure 3A,B). The partial suppression could be due to differences in promoter strength or regulation of secretion from other sensory neurons. Similar results were obtained when a functional *bbs-7* cDNA was expressed in a *bbs-7* mutant: two independently generated transgenic lines showed partial but highly significant reductions of the enhanced insulin secretion of *bbs-7* mutants when expressed exclusively in ADL (Figure 3C,D). Finally, use of a heat shock inducible promoter to induce expression of wild-type *bbs-7* in late fourth larval stage *bbs-7* mutants when the developmental programs for ciliated neurons have been completed led to a partial but significant abrogation of enhanced secretion (Figure 3E,F). Thus, the requirement of the BBSome for wild-type secretion is, in part, cell autonomous and not a consequence of altered neural development.

### Increased Neuroendocrine Release of *bbs* Mutants Depends on the Conserved Rab27/AEX-6 Exocytosis Pathway

As the cilia and BBS proteins have been shown to regulate transcription through the TCF/LEF1 transcription factor [29], we investigated whether the observed enhanced secretions could be secondary consequences of increased transcription in *bbs* mutants. While expression of FLP-21-mCherry translational reporter fusion using the ADL-specific *srh-220* promoter resulted in increased accumulation of the secreted FLP-21-mCherry in coelomocytes (Figure 1H), expression levels of the *Psrh-220::gfp* transcriptional reporter were similar in wild type and *bbs-9* mutants (Figure S3A,B). Furthermore, transcript levels in several *bbs* mutants, as measured by semi-quantitative RT-PCR, did not show any significant increase in transcription of *srh-220*, *daf-28*, *flp-21*, *daf-7*, or several other neuropeptide genes endogenously expressed in ADL and ASI neurons (Figure S3C). Thus, enhanced secretion levels of insulin, TGF-β ligand, and neuropeptides and the corresponding increase in their associated signaling pathways are unlikely to be secondary consequences of enhanced transcription of insulin and neuropeptide genes.

**Figure 4:**
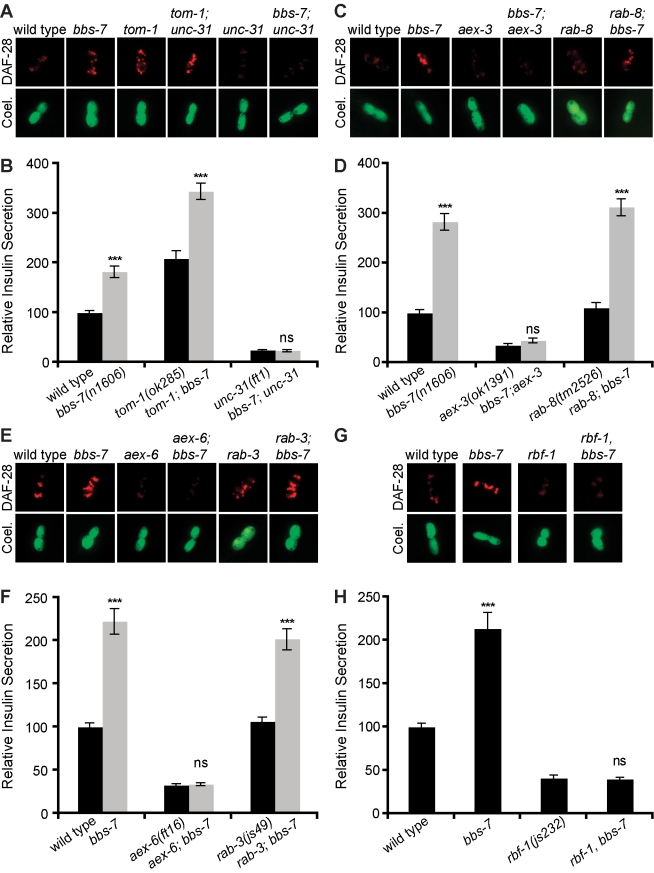
Increased secretion of *bbs* mutants requires the Rab27/AEX-6 dense-core vesicle exocytosis machinery. (A, C, E, G) Representative image of tagged insulin accumulation in coelomocytes, and (B, D, F, H) corresponding quantitation with standard error are shown (*** *p*<0.001; *ns*, not significant, comparing double mutants with the corresponding single mutant) for various components of dense-core vesicle transport and release.

To better understand the enhanced secretions of *bbs* mutants, we next investigated the requirements of various components of the dense-core secretion machinery. Regulated release of dense-core vesicles is triggered by Ca^+2^ entry leading to activation of UNC-31/CAPS, which promotes vesicle fusion with the plasma membrane [30]. We previously showed that UNC-31 is required for insulin release from ADL [21]. Therefore, we investigated whether enhanced secretion of dense-core vesicle contents seen in *bbs* mutants was dependent on UNC-31. We found that secretion levels of *bbs-7*; *unc-31* double mutants were indistinguishable from *unc-31* mutant alone (Figure 4A,B), indicating that the BBSome is a negative regulator of Ca^+2^ stimulated release of dense-core vesicles and does not affect the constitutive basal release seen in *unc-31* mutants. UNC-31 acts in parallel to TOM-1, the *C. elegans* tomosyn [26]. Tomosyns negatively regulate both dense-core and synaptic vesicle fusions by binding to and inhibiting syntaxins, an essential component of the vesicle fusion machinery [26]. As in *bbs* mutants, *tom-1* mutants exhibited increased secretion from ADL (Figure 3A,B) and the *tom-1* and *bbs-7* secretion phenotypes were additive (Figure 4A,B). The finding that loss of *unc-31* fully abrogated the hypersecretion of *bbs* mutants cannot distinguish whether the BBSome regulates secretion through mechanisms that directly modulate the *unc-31* pathway from indirect mechanisms that ultimately depend on UNC-31 mediated secretion. However, since the hypersecretion of *bbs* mutants was fully abrogated by *unc-31* but additive with that of *tom-1* mutants, these findings suggest that UNC-31 and the BBSome act in a common release pathway in parallel to TOM-1.

Dense-core vesicles are trafficked from their site of formation in the Golgi to the plasma membrane by Rab GTPases and their regulators. Rab GTPases are a highly conserved family of proteins involved in various aspects of vesicle fusion and transport. Previous studies have indicated that the BBSome regulates Rab8 localization and activity in mammalian cells [4]. *bbs* and *rab-8* mutants in *C. elegans* show similar defects in cilia membrane morphology, suggesting that the BBSome is also likely to regulate Rab8 activity in *C. elegans* [31]. However, *rab-8* mutants exhibited wild-type levels of secretion from ADL and loss of *rab-8* did not change the hypersecretion phenotype of *bbs-7* mutants (Figure 4C,D). This suggests that the BBSome might regulate dense-core vesicle secretion through different Rabs. Previous studies have suggested that Rab3 and Rab27 regulate vesicular exocytosis by promoting the movement and tethering of dense-core vesicles to the plasma membrane [32],[33]. We found that AEX-6, the *C. elegans* homolog of Rab27, was essential for dense-core vesicle secretion from ADL and that its loss abrogated the enhanced secretion of *bbs* mutants (Figure 4E,F). By contrast, loss of *rab-3*, another GTPase implicated in exocytosis, had no effect (Figure 4E,F). The enhanced secretion of *bbs* mutants was also dependent on rabphilin/RBF-1, an effector of RAB-27/AEX-6 (Figure 4G,H), and AEX-3, a RabGEF previously shown to regulate RAB-27/AEX-6 and RAB-3 (Figure 3C,D) [32]. Taken together, these data are consistent with a model whereby the BBSome acts as a negative regulator of AEX-6/RBF-1/UNC-31, the worm counterpart of mammalian Rab27/rabphilin/CAPS exocytosis machinery. Given that the mammalian BBSome binds to Rabin8, a RabGEF for Rab8, and regulates Rab8 activity [4], the BBSome might regulate dense-core vesicle secretion by regulating the activity of AEX-3, the RabGEF for AEX-6/Rab27.

To further investigate the effects of *bbs* deficiency on dense-core vesicles, we examined subcellular localization of IDA-1-GFP, a tyrosine phosphatase-like receptor involved in protein secretion that has been used as a maker of dense-core vesicles [34]. The overall expression levels of IDA-1-GFP was similar in wild type and *bbs* mutants, and in both cases GFP puncta were clearly visible along axons as well as dendrites terminating in cilia (Figure S5 and unpublished data). While the IDA-1-GFP reporter is largely excluded from the cilia of wild type animals, it was prominently visible in those of *bbs* mutants (Figure S5). Presence of the marker in cilia of *bbs* mutants could suggest either mis-sorting of proteins normally localized to dense-core vesicles or the appearance of intact dense-core vesicles, which are normally excluded from cilia, within these structures.

One mechanism that has been proposed to ensure proper segregation of cilia-localized membrane proteins from other plasma membrane proteins is the proteinaceous structure at the base of the cilia known as the transition zone [35]. The transition zone is thought to provide a barrier that helps exclude non-ciliary proteins from the cilia [35]. Mutations in components of the transition zone underlie ciliopathies such as Merkel-Gruber syndrome and nephronophthisis, which share some clinical features such as renal abnormalities with Bardet-Biedl syndrome [2]. Unlike *bbs* mutations, however, mutations in genes encoding components of the transition zone, such as *mksr-1* and *mksr-2* did not cause enhanced neuroendocrine secretion (Figure S2).

### Hyperactive Neuroendocrine Signaling Does Not Cause Cilia Defects in *bbs* Mutants

The finding that loss of BBSome components enhances secretion of dense-core vesicles prompted us to examine the relationship between hyperactive neuroendocrine signaling and ciliary defects of *bbs* mutants. Since losses of various IFT components result in reduced secretion, we first investigated the requirement of functional cilia for the enhanced secretion of *bbs* mutants. We found that mutations in each of *che-2* and *che-11*, which cause reduced secretion levels (Figure 1A,B), completely abrogate the enhanced secretion phenotype of *bbs* mutants (Figure S2). These findings further validate the notion that the excess secretion phenotype of *bbs*-deficient animals is not simply a consequence of defective cilia but rather that some level of normal ciliary function is required in order for the secretion phenotype of *bbs* mutants to be manifested.

We next investigated whether ciliary defects of *bbs* mutants were dependent on hypersecretion of dense-core vesicles. To assess structural integrity of cilia in various mutants, we used a dye-filling assay. Subsets of *C. elegans* sensory neurons have their ciliated endings located near the surface of the animals and are directly exposed to the environment for chemosensation. Upon immersion of whole animals in a fluorescent dye solution, these neurons become visible as the dyes can enter these neurons through their cilia (Figure S1) [36]. Since animals with defective cilia formation such as the IFT mutants fail to dye fill (Figure S1B) [36], this assay has been used to assess cilia integrity and proper opening of the cilia to the external environment. As previously reported, we found that BBSome mutants also failed to dye fill (Figure S1) [18],[37],[38]. Interestingly, while mutations in the Rab27/rabphilin/CAPS exocytosis machinery abrogated the enhanced secretion of *bbs* mutants, they did not restore structural integrity to cilia in these animals as judged by the inability of the double mutants to dye fill (Figure S1C,D). These findings suggested that the structural and functional defects of cilia in *bbs* mutants are unlikely to be a consequence of hypersecretion of dense-core vesicles.

**Figure 5:**
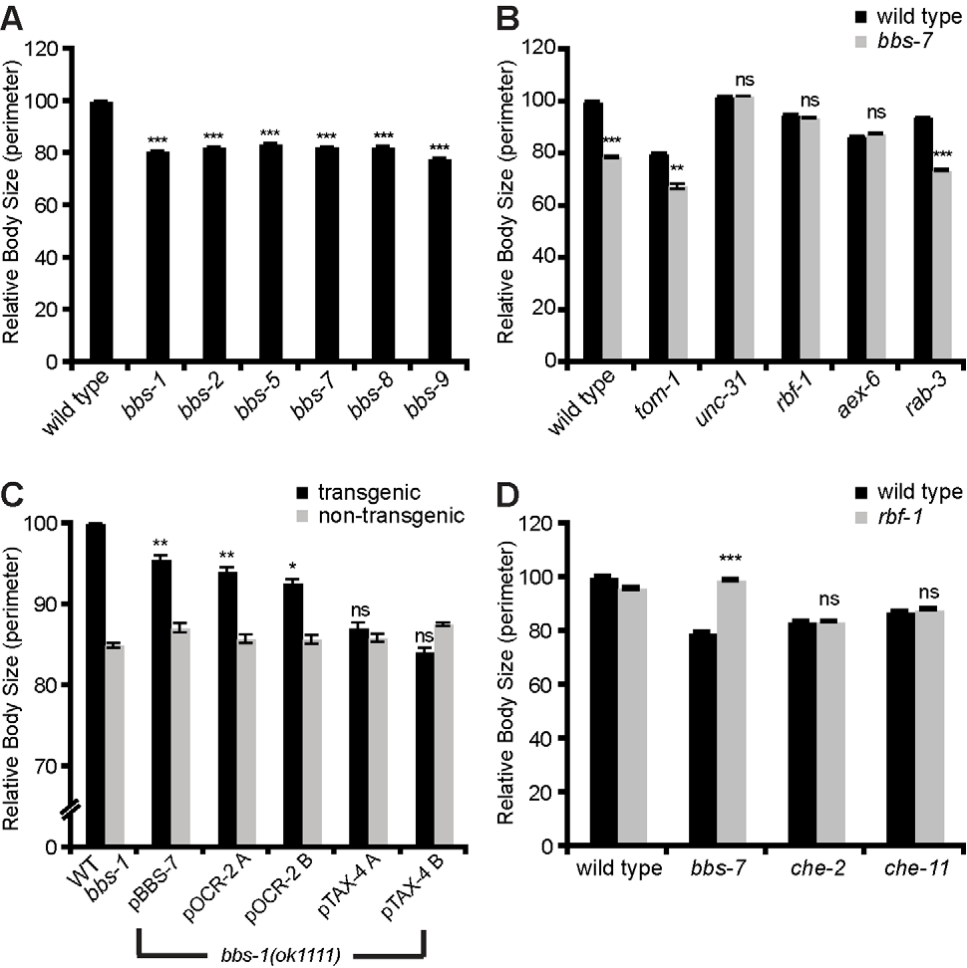
Hyperactive secretion causes small body size of *bbs* mutants. (A) Quantitation with standard error of body sizes of *bbs* mutants compared to wild type (*** *p*<0.001). (B) Mutations that abrogate enhanced secretion (*rbf-1*, *aex-6*, *unc-31*) normalize while loss of *tom-1* further reduces the body size of *bbs* mutants (*** *p*<0.001, ** *p*<0.01; *ns*, not significant, comparing double mutants to the corresponding single mutant). (C) Quantitation with standard error of *bbs-1* mutants expressing *bbs-1* cDNA under the indicated neuron-specific promoters. Expression patterns of these promoters are listed in Table S1 (* *p*<0.05, ** *p*<0.01, *** *p*<0.001, comparing transgenic animals to non-transgenic siblings). (D) Rabphilin, *rbf-1*, suppresses the body-size defect of *bbs-7* but not of IFT mutants, *che-2* and *che-11* (*** *p*<0.001; *ns*, not significant, comparing double mutant to *bbs-7* or IFT single mutant).

To determine if ciliary structural deficiencies that manifest as dye-filling defects are a pre-requisite for hyperactive secretion, we examined the hypersecretion *tom-1* mutants and found that they exhibited normal dye filling (Figure S1B). We also found that while *bbs-5* mutants display similarly enhanced levels of secretion as other BBSome mutants (Figures 1C,D and 5A), they dye fill like wild-type animals (Figure S1A). Additionally, *bbs-5* mutants displayed normal expression of the serotonin synthesis enzyme, *tph-1* (Figure S4C,D), whereas expression of this enzyme was shown to be elevated in many cilia-defective mutants [39] (Figure S4C,D), further suggesting the presence of functional cilia, in *bbs-5* mutants.

Together these findings suggest that the enhanced secretion phenotype of *bbs* mutants depends on retention of some level of ciliary function and that ciliary structural defects are not a pre-requisite for enhanced release of dense-core vesicles. In turn, enhanced release of dense-core vesicles can be abrogated without concomitant correction of ciliary structural abnormalities of *bbs* mutants.

### The Reduced Body Size of *bbs* Mutants Is Due to Enhanced Secretion

As various organism-wide phenotypes are controlled by neuroendocrine signals, we asked whether some of the phenotypes seen in *C. elegans bbs* mutant might be due to enhanced neuroendocrine signaling in these mutants. Human BBS patients are short statured, *bbs* mutant mice are born small [13],[14],[40], and *bbs* mutant *C. elegans* have a reduced body size (Figure 5), suggesting that size regulation might be a conserved function of the BBSome. We found that *tom-1* mutants, similar to BBSome-deficient animals, exhibited reduced adult body size (Figure 5B), suggesting that hypersecretion without ciliary defects can cause small body size. Considering that some IFT mutants also exhibit small body sizes (Figure 5D) [41], we sought to distinguish whether the small body size of *bbs* mutants could be attributed to either hypersecretion or ciliary defects. To do so, we suppressed the enhanced secretion of BBSome mutants with mutations in the dense-core vesicle exocytosis pathway (Figure 5B). Loss of *rbf-1*, which suppressed the enhanced dense-core vesicle release of *bbs* mutants, fully restored normal body size to *bbs-7* mutants (Figure 5B,D). By contrast, *rbf-1* mutation failed to change the small body of IFT mutants, suggesting that IFT and *bbs* mutants regulate size through different pathways (Figure 5D). Similarly, loss of *unc-31* and *aex-6*, but not that of *rab-3*, conferred nearly wild-type body size to *bbs-7* mutants (Figure 5B). Importantly, in all of these cases normalizations of body sizes were achieved without functional correction of ciliary defects, as the double mutants remained dye-filling defective (Figure S1C,D, unpublished data). Furthermore, *tom-1* mutants that exhibited an additive secretion phenotype with BBSome mutants caused a further reduction in the size of *bbs* mutant (Figure 5B). Together, these findings suggest that the small size of BBSome mutants is due to enhanced secretion of dense-core vesicle cargoes and distinct from IFT mutants.

In further support of the notion that distinct mechanisms underlie the small sizes of BBSome and IFT mutants, we also found that neurons that function in size regulation in *bbs* mutants are distinct from the neurons that function in size regulation in IFT mutants. Normal body size can be restored to *che-2* IFT mutants by expressing wild-type *che-2* cDNA in a subset of ciliated neurons using the *tax-4* promoter [42]. By contrast, expression of functional *bbs* cDNAs using a *tax-4* promoter failed to alter the small body size of *bbs* mutants (Figure 5C and Table S1). The body-size phenotype of *bbs* mutants could, however, be reverted to wild-type levels when *bbs* cDNAs were expressed using an *ocr-2* promoter, which directs expression to a distinct subset of neurons than those expressing *tax-4*. This result indicates the spatial requirements of the IFT machinery and the BBSome can be attributed to distinct, non-overlapping neurons in the regulation of body size (Figure 5C, see Table S1 for expression pattern). Thus, despite phenotypic similarities, the size phenotypes of BBSome and IFT mutants are based on distinct cellular and molecular mechanisms.

### Enhanced Neuroendocrine Secretion Underlies Metabolic Phenotypes of *bbs* Mutants

**Figure 6:**
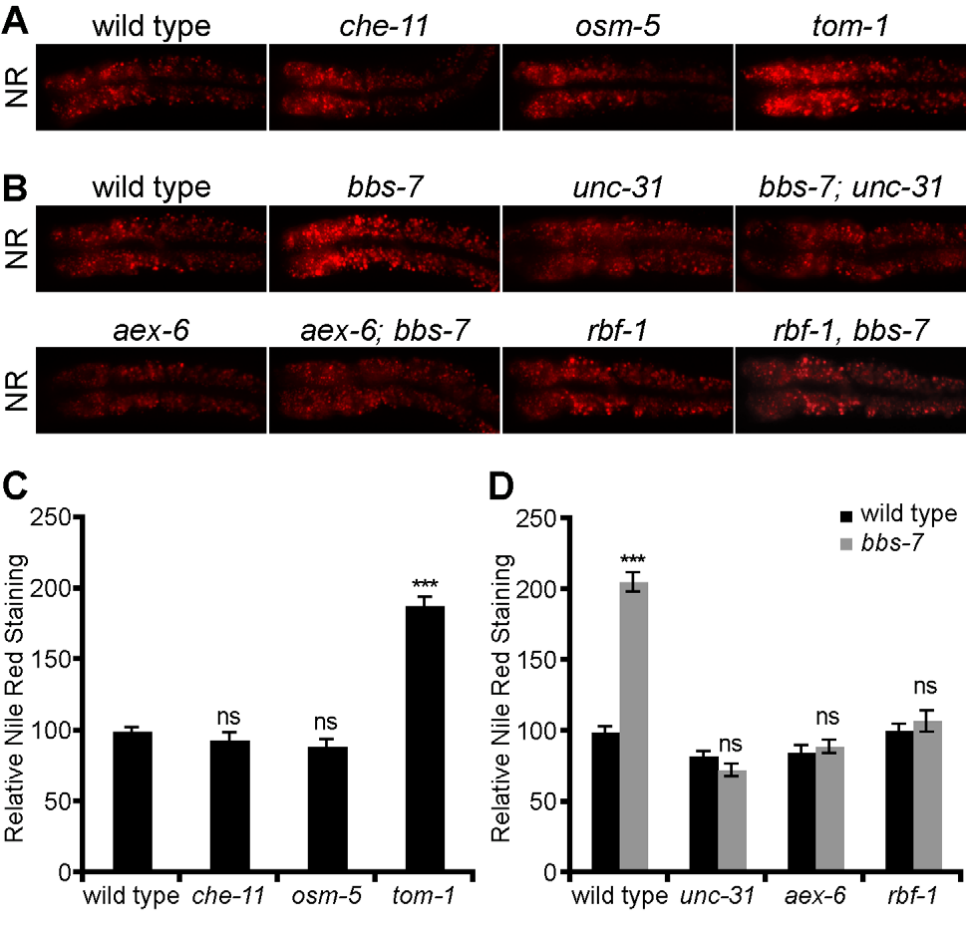
Enhanced secretion caused metabolic defect in *bbs* mutants. Representative images (A, C) and quantitation with standard error (B, D) of Nile Red staining in the first pair of intestinal cells of *bbs-7*, IFT, and dense-core vesicle secretion mutants (*** *p*<0.001; *ns*, not significant, compared to wild type in B or comparing double mutants to the corresponding single mutant in D).

As in the case of small body size, we found that a metabolic phenotype exhibited by *bbs* mutants is mimicked by the hyperactive secretion mutant *tom-1* but not by IFT mutants. Specifically, *bbs* mutants were previously reported to exhibit increased accumulation of Nile Red and bodipy-labeled fatty acids, vital dyes used as proxies for assessment of metabolic state in intact animals [17],[38],[43]–[45]. As in body size, *tom-1* mutants exhibited a Nile Red phenotype that was reminiscent of that seen in BBSome mutants, while IFT mutants such as *osm-5* and *che-11* exhibited wild-type patterns of Nile Red staining (Figure 6A,C). As in body size and release of dense-core vesicles, *tom-1* and *bbs-7* mutants exhibited additive Nile Red phenotype (Figure S4A,B). Furthermore, mutations in the Rab27 exocytosis pathway, which abrogated the enhanced secretion of *bbs* mutants, restored wild-type levels of Nile Red staining to *bbs-7* mutants (Figure 6B,D), suggesting that enhanced secretion underlies the Nile Red phenotype of *bbs* mutants.

### Enhanced Serotonin Release Underlies the Feeding Phenotypes of *bbs* Mutants

**Figure 7:**
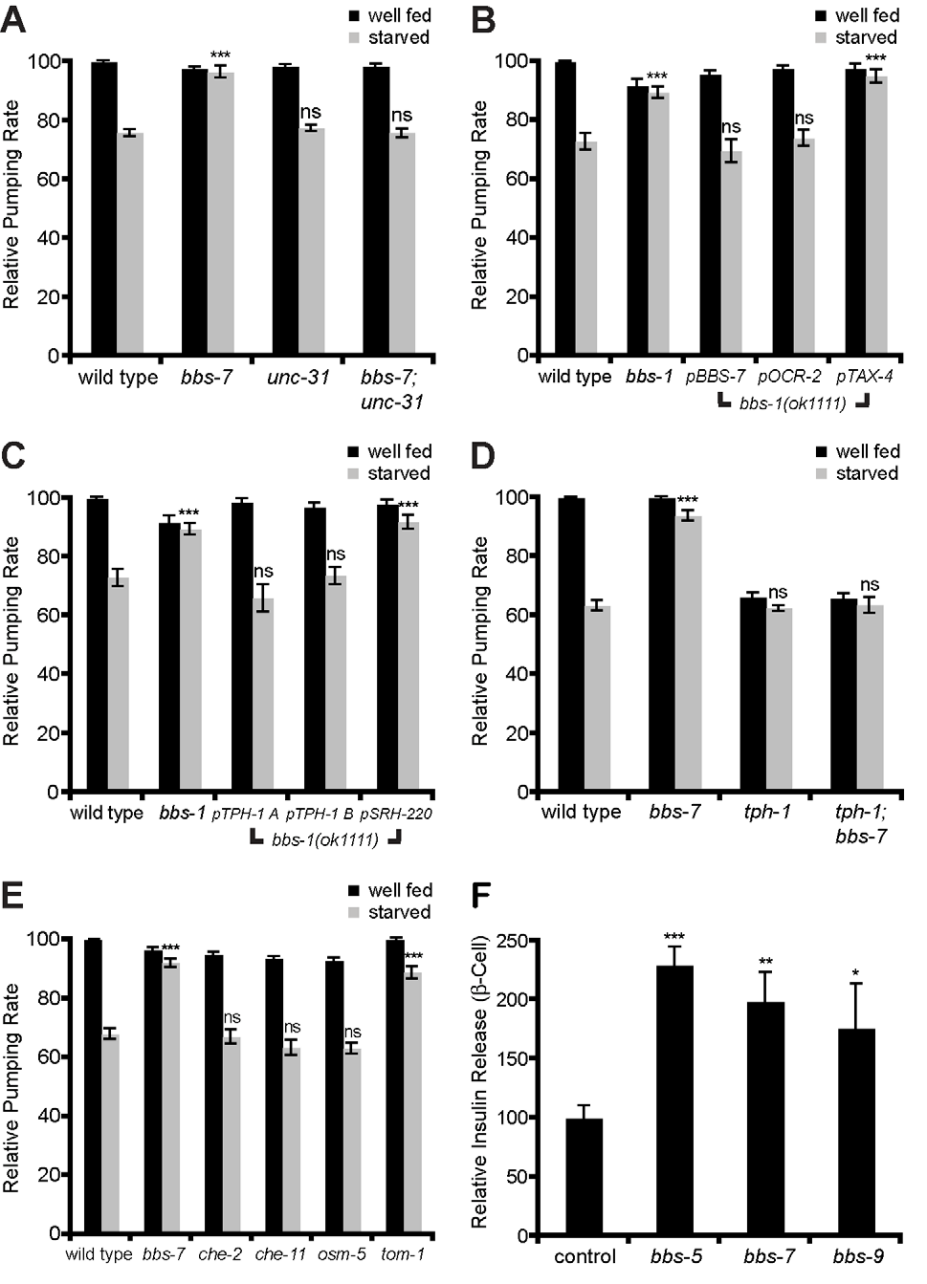
Reduction of dense-core vesicle secretion corrects feeding abnormalities of *bbs* mutants and BBS siRNA enhanced insulin secretion from β-cells. *unc-31*, secretion defective mutant (A) and *tph-1* serotonin synthesis mutant (D) suppress the increased pumping rate of *bbs* mutants when starved (*** *p*<.001; *ns*, not significant, compare to wild-type starved pumping rate). (B, C) The pumping rate of transgenic animals expressing *bbs-1* cDNA under the indicated neuron-specific promoters in *bbs-1* mutants was determined. Expression patterns of these promoters are listed in Table S1. Pumping rate was restored to wild-type level when *bbs-1* cDNA was expressed in the sole serotonergic ciliated neuron, ADF (*** *p*<.001; *ns*, not significant, compared to wild-type starved pumping rate). (E) *bbs* mutants shared increased pumping rate when starved with hypersecretion *tom-1* mutants in contrast to *che-2*, *che-11*, and *osm-5* IFT mutants (*** *p*<.001; *ns*, not significant, compared to wild-type starved pumping rate). (F) siRNA against BBSome subunits in Min6 cells increases release of insulin into the surrounding media relative to control siRNA (* *p*<0.05, ** *p*<0.01, *** *p*<.001, compared to control siRNA).

In mice, the obesity seen in the setting of *bbs* deficiency has been attributed to hyperphagia [13]. *C. elegans* BBSome mutants similarly showed an altered food intake behavior (Figure 7). Food intake behavior in *C. elegans* is assessed by counting the pumping rate of the pharynx, an organ for ingesting bacteria [16],[46]. Pumping rate is modulated by the availability of food supplies, food quality, and prior feeding experience of the animals [47]. Although under plentiful food conditions, wild type and *bbs*-deficient *C. elegans* display similar pumping rate [38], we found that *bbs*-deficient animals, unlike wild-type animals, continue to exhibit rapid pharyngeal pumps even when food supplies are exhausted (Figure 7). This rapid pumping rate is unlikely to be merely a consequence of defective cilia as many other cilia-defective mutants exhibit wild-type pumping rate in the presence or absence of food (Figures 7E, S4C) [41]. As in the case of body size and vital dye staining patterns, reducing secretion of dense-core vesicles without concomitant correction of ciliary defects was sufficient to restore wild-type rates of food intake in *bbs* mutants (Figure 7A).

One molecular mechanism known to modulate pumping rate is serotonin signaling [48]. In *C. elegans* as in mammals, serotonin production is dependent on the tryptophan hydroxylase enzyme, *tph-1*. This enzyme is expressed in only a few neurons, only one pair of which, the ADF neurons, is ciliated [48]. To investigate whether abnormal serotonin signaling may underlie the enhanced pumping rate of food-deprived *bbs* mutants, we expressed functional *bbs-1* cDNA using a *tph-1* promoter in *bbs-1* mutants. *bbs-1* reconstitution was sufficient to fully restore wild-type pumping rate to *bbs-1* mutants (Figure 7B, Table S1). Similarly, all other promoters used to reconstitute wild-type *bbs-1* that express in ADF neurons fully rescued the feeding phenotype, whereas expression of *bbs-1* using promoters that do not target ADF failed to alter the enhanced pumping rate of *bbs-1* mutants (Figure 7B,C, see Table S1 for expression pattern). Similar results were obtained when *bbs-7* cDNA was expressed in the ADF neurons of *bbs-7* mutants (Table S1). Consistent with the rescue data, loss of *tph-1* fully abrogated the enhanced pumping rate of *bbs* mutants (Figure 7D). These findings suggest that the elevated pumping rate of BBSome mutants is due to excessive serotonin signaling initiated from ADF neurons.

Since elevated expression of *tph-1* in ADF neurons has been reported in numerous cilia-defective mutants including *bbs* (Figure S4C,D) [39], we sought to distinguish whether the excess serotonin signaling of *bbs*-deficient animals could be attributed to excessive production of serotonin or to increased release of this biogenic amine through dense-core vesicles. Despite elevated *tph-1* expression, cilia-defective mutants such as *che-2*, *che-11* and *osm-5* exhibited wild-type pumping rates in the presence or absence of food (Figures 7E, S4C), suggesting that increased *tph-1* expression does not necessarily correlate with excessive serotonin signaling. In contrast, the hypersecretion mutant *tom-1* mimicked the *bbs* mutant feeding phenotype in that *tom-1* mutants exhibited elevated pumping rate when removed from food (Figure 7E). Furthermore, *bbs-5* mutants, in which cilia are relatively normal as assessed by dye filling (Figure S1A), exhibited wild-type level of *tph-1* expression in ADF neurons yet displayed elevated pumping in the absence of food (Figure S4C,D). Thus, the elevated pumping rate of *bbs* mutants is likely due to excessive release of serotonin rather than increased synthesis via elevated expression of *tph-1*.

Taken together, our data indicate that while some ciliary-defective mutants display size, metabolic, and feeding phenotypes that are reminiscent of those seen in the *bbs* mutants, the underlying molecular and cellular bases of these phenotypes are likely to be distinct. In turn, mutations that cause enhanced secretion of dense-core vesicles without gross structural defects of cilia mimic several physiological phenotypes seen in *bbs* mutants.

### Inactivation of BBSome Components Causes Increased Insulin Secretion from Mammalian β-Cells

Increased levels of circulating leptin and insulin in *bbs*-deficient mice and humans have been reported [1],[11],[14],[49]. In the case of insulin, the increase is generally assumed to be a secondary consequence of obesity and insulin resistance. However, our data raise the possibility that elevated levels of these circulating hormones may be in part a consequence of enhanced dense-core secretion rather than secondary outcomes of obesity. To further support this possibility, we employed Min6 cells, a mouse pancreatic β-cell line that is ciliated and expresses BBSome components. Treatment of Min6 cells with siRNAs targeting each of three distinct BBSome subunits, Bbs5, Bbs7 and Bbs9, resulted in ∼1.5–2-fold increase in secreted insulin compared to control siRNA (Figure 7F). Importantly, since these experiments were conducted in cell lines, the enhanced secretion could not be merely attributed to a secondary consequence of organismal obesity. Consistent with this interpretation, excess circulating level of leptin is evident in *bbs*-deficient mice before the onset of weight gain [11].

## Discussion

Although disruptions in either the IFT machinery or the BBSome can lead to similar structural and functional defects, here we show that they elicit opposite effects on dense-core vesicle secretion. Specifically, defects in the BBSome cause elevated secretions of dense-core vesicles, while IFT defects generally cause reduced secretions. Our findings indicate that this excess secretion is largely cell autonomous, is not a consequence of altered development, and is dependent on the *C. elegans* counterpart of Rab27/rabphillin/CAPS exocytosis machinery but distinct from the Rab8/Rabin8 vesicular transport machinery that helps target membranes and cargoes to the cilium [4]. Therefore, we propose that the role of the BBSome complex in vesicular transport within ciliated cells should be expanded from ciliary functions [4],[5],[8],[10],[18],[19] and melanosome movement [4],[50] to also include dense-core vesicle exocytosis.

The current paradigm for understanding the myriad manifestations of *bbs* deficiency is largely framed in the context of defective ciliary functions arising from a failure to properly sort receptors and other signaling molecules to the cilia [5],[6],[8],[10],[11],[18],[19]. As such, mutations in the IFT machinery and the BBSome are thought to cause similar ciliary defects albeit with different levels of severity. This view emerged, in part, by the observations that mutations in IFT components result in organism-wide phenotypes that resemble those of BBSome deficiency [12]. Our findings here indicate that while *bbs* deficient animals share some of the defects of IFT mutants, the consequences of these mutations on dense-core vesicle secretion and resultant phenotypes are dramatically different from one another. More importantly, our findings challenge the view that similar mechanisms underlie all of the phenotypic similarities caused by BBSome and IFT mutants. For instance, while mutants in both IFT and BBSome components share defects in body size, proper body size regulation requires these components in distinct subsets of sensory neurons. Moreover, the small body sizes of *bbs* mutants could be reverted to wild type upon abrogation of enhanced secretion of dense-core vesicles, while similar manipulations had no effects on the small body sizes of IFT mutant. Similarly, it has been reported that BBSome and IFT mutants displayed distinct phenotypes of a specific learning behavior in *C. elegans* [51]; our findings suggested that this difference is likely to reflect a role of dense-core vesicle release in this behavior. Finally, consistent with the notion that some of the phenotypic consequences of *bbs* deficiency are due to hypersecretion of dense-core vesicles, we found that *tom-1* mutants, which cause hypersecretion without obvious ciliary defects, exhibit the size, metabolic, and feeding abnormalities similar to *bbs* mutants.

We do not currently know the precise mechanisms through which *bbs* deficiencies cause hypersecretion of dense-core vesicles. Although the enhanced secretion phenotype of *bbs* mutants can be manipulated independently of their ciliary defects, we found that wild type IFT activity is required for manifestation of the hypersecretion phenotype. These findings are consistent with several models. In one model, *bbs* mutants may mislocalize or excessively accumulate receptors that modulate dense-core vesicle release within the cilia [9]. Given that cilia bound vesicles and dense-core vesicles both arise at the trans-Golgi membrane, it is possible that as a coat-protein, the BBSome not only sorts the appropriate cilia-localized proteins but also prevents the inappropriate accumulation of other molecules in this organelle. Consistent with this scenario, we found that accumulation of IDA-1-GFP, a marker of dense-core vesicles normally excluded from the cilia, accumulates in cilia of *bbs* mutants (Figure S5). An alternative but not mutually exclusive possibility is that the BBSome may regulate dense-core vesicle release as a separate function from its role in sorting membranes and cargoes to the cilium. Given that the BBSome regulates cilia-bound vesicles through binding Rabin8 [4], it is possible that the BBSome regulates the Rab27/rabphillin/CAPS exocytosis pathway through binding specific regulatory components of this machinery such as AEX-3, the RabGEF for RAB-27/AEX-6. The idea that the transport machinery associated with cilia formation and function may not be restricted to these organelles and may have evolved from existing cellular machinery has been suggested by the recent demonstration that IFT proteins also play a role in polarized receptor trafficking to a cellular region reminiscent of the cilia in non-ciliated cells [52]. Additionally, the BBSome mediates the movement of melanosomes on microtubules tracks, an organelle not directly associated with the cilia, suggesting that the BBSome might have cilia-independent functions [50]. The genetic epistasis studies presented here do not distinguish between these possibilities. Whether the effects of *bbs* deficiency on dense-core vesicle secretion are cilia-independent functions of the BBSome or secondary consequences of ciliary abnormalities, our findings challenge the existing paradigm that phenotypic manifestations of the *bbs* deficiency are recapitulated by IFT mutations. The most salient insight to emerge from our findings is that several of the organismal phenotypes seen in *bbs* mutants are shared with hypersecretion *tom-1* mutants and can be reverted to wild-type by abrogating the hypersecretion of these mutants without concomitant correction of ciliary defects. Although a number of defects seen in *bbs* patients are likely to be consequences of mis-localized ciliary proteins, we believe that the etiologies of some features of BBS merit re-evaluation in light of the role of this complex in dense-core vesicle secretions. While the specific hormonal pathways that underlie the behavioral and physiological abnormalities of *C. elegans* may or may not play similar roles in mammals, excessive hormonal secretions are likely to contribute to disease manifestations in BBS patients. For instance, obesity and hyperphagia of BBS patients may be a direct consequence of or exacerbated by increased secretion of appetite-promoting hormones, and hypertension in these patients could be caused by increased release of catecholamines. The finding that hypersecretion in *bbs* deficiency can be abrogated without correcting concomitant ciliary defects may open the door to new strategies for treating patients with Bardet-Biedl syndrome.

## Materials and Methods

### Strain Constructions

Strains were constructed by standard *C. elegans* methods [53]. Strains obtained from the *C. elegans* Genetics Center or the National Bioresourse Project were backcrossed at least 4× with wild type before phenotypic assessment. Transgenic animals were generated by injecting the plasmid of interest at 100 ng/µl with 20–50 ng/µl of *Punc-122::GFP* or *Pmyo-3::GFP* as co-injection marker. Plasmids were construct by Gateway cloning as described in [21].

### Coelomocyte Insulin and Neuropeptide Uptake Assay

Coelomocyte uptake assays were performed as described in [21] with the exception that 20–40 animals were imaged for each strain at sub-saturating exposure with the shutter half open to split fluorescence between the eyepiece and the camera.

### *Ptph-1::GFP* and *Psrh-220::GFP* Measurements

∼30 sychronized adults for *Ptph-1::GFP* and L4 of *Psrh-220::GFP* expressing animals were imaged at 40× magnification on a Zeiss Axioplane microscope at a sub-saturating exposure. The neuron of interest was circled and GFP fluorescent within that neuron quantified with OpenLab software. The minimum fluorescence is subtracted from the mean for each cell. The mean and standard error of the mean was determined for each strain and and normalized to wild type.

### Heat Shock Induction

Synchronized mid L4 animals were heat shocked on plates at 37°C for an hour and allowed to recover at 20°C for 4 h before phenotypic assessment.

### Body Size Measurement

DIC images of 20–40 animals were taken at 5× magnification and the outline of each animal was traced by OpenLab software. The mean and standard error of the perimeter was calculated for each strain and normalized to wild type.

### Dauer Assay

∼200 synchronized L1 animals for each strain were plated onto five plates and incubated at 25°C for 2 d before the percentage of dauer animals was determined along with standard error of the mean.

### Social Feeding Assay

The assay was preformed as described in [28] on six plates per strain. The percentage of animals feeding in groups was determined along with standard error of the mean.

### Dye-Fill Assay

∼300 synchronized L4 worms were washed into 1.5 ml eppendorf with S-basal and incubated with 1 µl of 10 mg/µl of DiI in 1 ml of S-basal for ∼2 h rotating at room temp. Worms were than pelleted and washed once with S-basal and plated onto an OP50 seeded plate for ∼1.5 h before imaging at 16× on a Zeiss Axioplan microscope.

### Pharyngeal Pumping Assay

Pumping rate was measured essentially as described in [16] with the exception that pumping rates were determined in 10-s intervals for 10 animals for each genotype. The average pumping rate and standard error of the mean were determined and normalized to wild type.

### Nile Red Staining Assay

∼200 synchronized L1 animals were plated onto a 6 cm plate containing 0.05 µg/ml of Nile Red and incubated at 20°C for 3 d. Gravid animals were imaged using a TRITC filter at 16× magnification at a sub-satrating exposure with the shutter half open to split fluorescence between eyepiece and the camera on a Zeiss Axioplan microscope. The first pair of intestinal cells was traced using the DIC image in OpenLab and the mean Nile Red fluorescent minus the background was determined for 10–20 animals for each genotype. The average fluorescent along with the standard error of the mean was determined for each strain and normalized to wild type.

### siRNA and Insulin Release in Min6 Cells

Min6 cells were treated with siRNAs from Qiagen with Hiperfect (Qiagen) for 4 d in triplicates. Cells were rested in Krebs-Ringer bicarbonate buffer for 2 h and then allowed to release insulin into the media for an hour. Insulin was measured by an ELISA kit (Mercodia) in duplicates and normalized to the total insulin obtained after cell lysis. The mean and standard error was calculated and expressed as a percentage of the control siRNA.

### Semi-Quantitative RT-PCR

∼10,000 synchronized L1 for each genotype were grown to L4 at 20°C. RNA extraction, cDNA prepartion, and RT-PCR were preformed as described in [54] with primers and syber green PCR mix from Qiagen. The data were standardized to the actin gene, *act-1*. Data from two independent growths were aggragated for statistical analysis.

### Statistical Analyses

All statistical analyses were performed using a two-tailed student *t* test.

## Supporting Information

Figure S1 Dye-filling phenotype of BBSome mutants.
(PDF)

Figure S2 Increased secretion of BBSome mutants depends on IFT. Transition zone mutants do not increase secretion.
(PDF)

Figure S3 BBSome mutants have normal expression of neuropeptide genes.
(PDF)

Figure S4 Tomosyn and BBSome mutants have additive Nile Red phenotype. Pumping phenotype of BBSome mutants is not caused by increased expression of *tph-1*.
(PDF)

Figure S5 Dense-core vesicle marker, IDA-1, localized to cilia in *bbs* mutants.
(PDF)

Table S1 Tissue-specific rescue of *bbs* mutants.
(PDF)

## Abbreviations

GEF: guanine nucleotide exchange factor
IFT: intraflagellar transport
