# Supplementary material for: Hyperactive Neuroendocrine Secretion Causes Size, Feeding, and Metabolic Defects of C. elegans Bardet-Biedl Syndrome Mutants
Source: PLoS Biol. 2011 Dec 13;9(12):e1001219. doi: 10.1371/journal.pbio.1001219 (PMC3236739; doi:10.1371/journal.pbio.1001219)
Supplement: Figure S1 — Dye-filling phenotype of BBSome mutants. (PDF) [file pbio.1001219.s001.pdf]

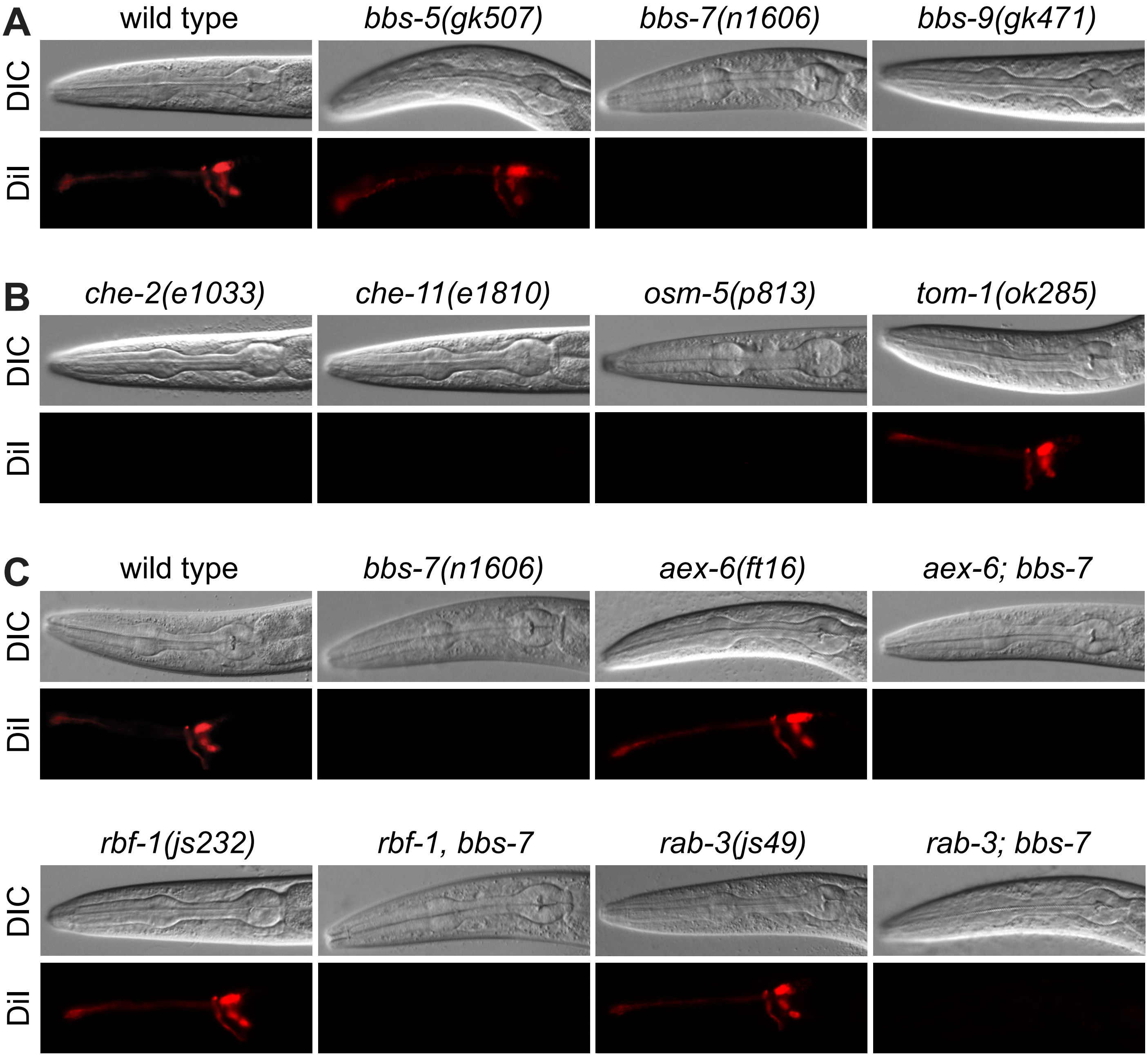

Supplemental Figure 1. Dye-Filling Phenotype of BBSome Mutants

(A) BBSome mutants other than *bbs-5* are dye-filling defective indicating defects in cilia structural integrity. (B) IFT but not hyperactive secretion *tom-1,* mutants are dye-filling defective. (C) Mutations that suppress the enhanced secretion of dense-core vesicle of *bbs-7* mutants: Rab27/*aex-6*, rabphilin/*rbf-1* do not suppress the dye-filling defect of *bbs-7* mutant.
