# Supplementary material for: Hyperactive Neuroendocrine Secretion Causes Size, Feeding, and Metabolic Defects of C. elegans Bardet-Biedl Syndrome Mutants
Source: PLoS Biol. 2011 Dec 13;9(12):e1001219. doi: 10.1371/journal.pbio.1001219 (PMC3236739; doi:10.1371/journal.pbio.1001219)
Supplement: Figure S2 — Increased secretion of BBSome mutants depends on IFT. Transition zone mutants do not increase secretion. (PDF) [file pbio.1001219.s002.pdf]

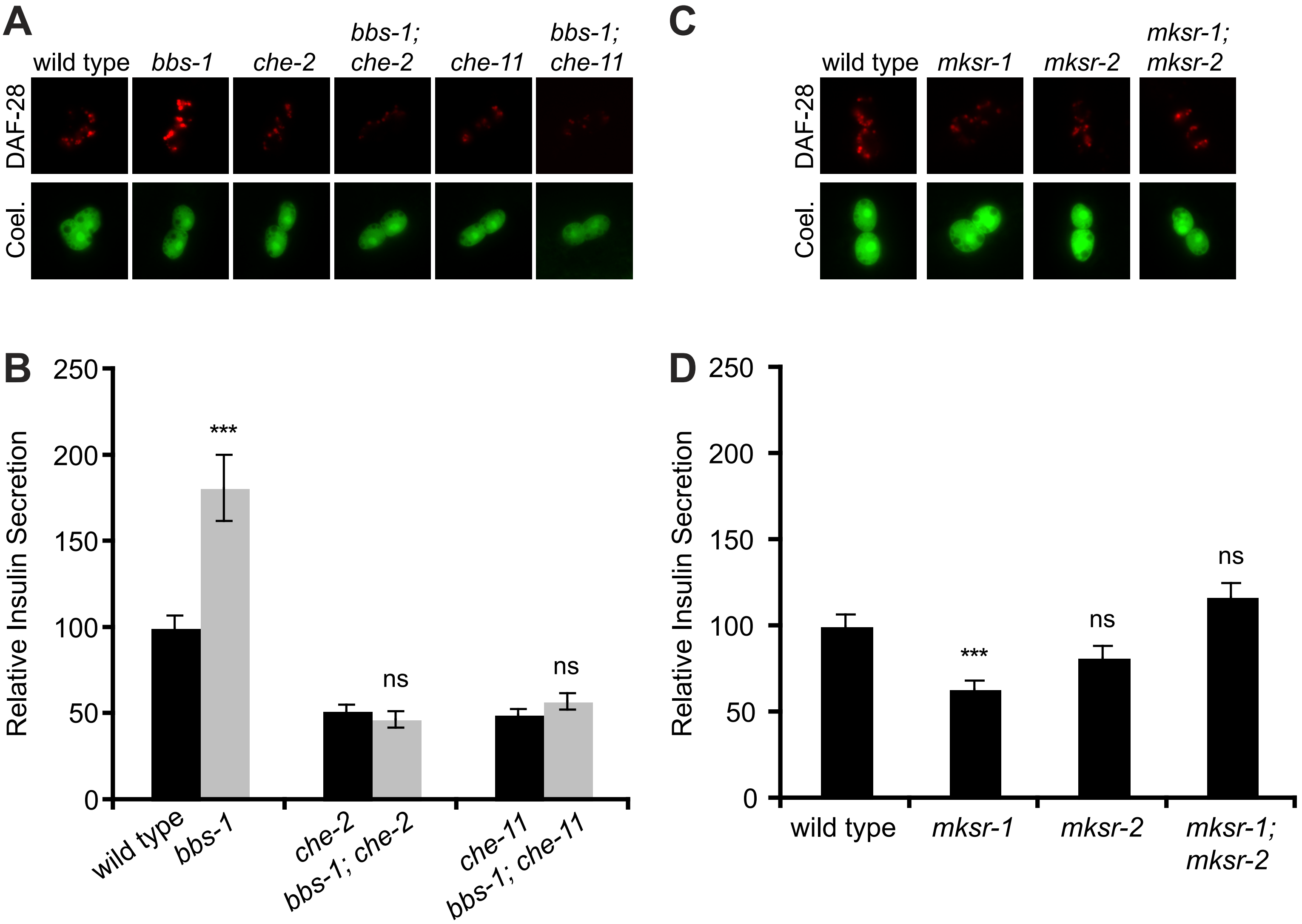

Supplemental Figure 2. Increased Serection of BBSome Mutant Depends on IFT. Transition Zone Mutants Do Not Increase Secretion.

Representative images (A, C) and quantitations with standard error (B, D) of insulin accumulation in (coel.) coelomocytes in IFT (A, B) and transition zone (C, D) mutants. (*** = p-value < 0.001, ns = not significant comparing single mutant to double mutant in B and to wild type in D).
