# Supplementary material for: Hyperactive Neuroendocrine Secretion Causes Size, Feeding, and Metabolic Defects of C. elegans Bardet-Biedl Syndrome Mutants
Source: PLoS Biol. 2011 Dec 13;9(12):e1001219. doi: 10.1371/journal.pbio.1001219 (PMC3236739; doi:10.1371/journal.pbio.1001219)
Supplement: Figure S3 — BBSome mutants have normal expression of neuropeptide genes. (PDF) [file pbio.1001219.s003.pdf]

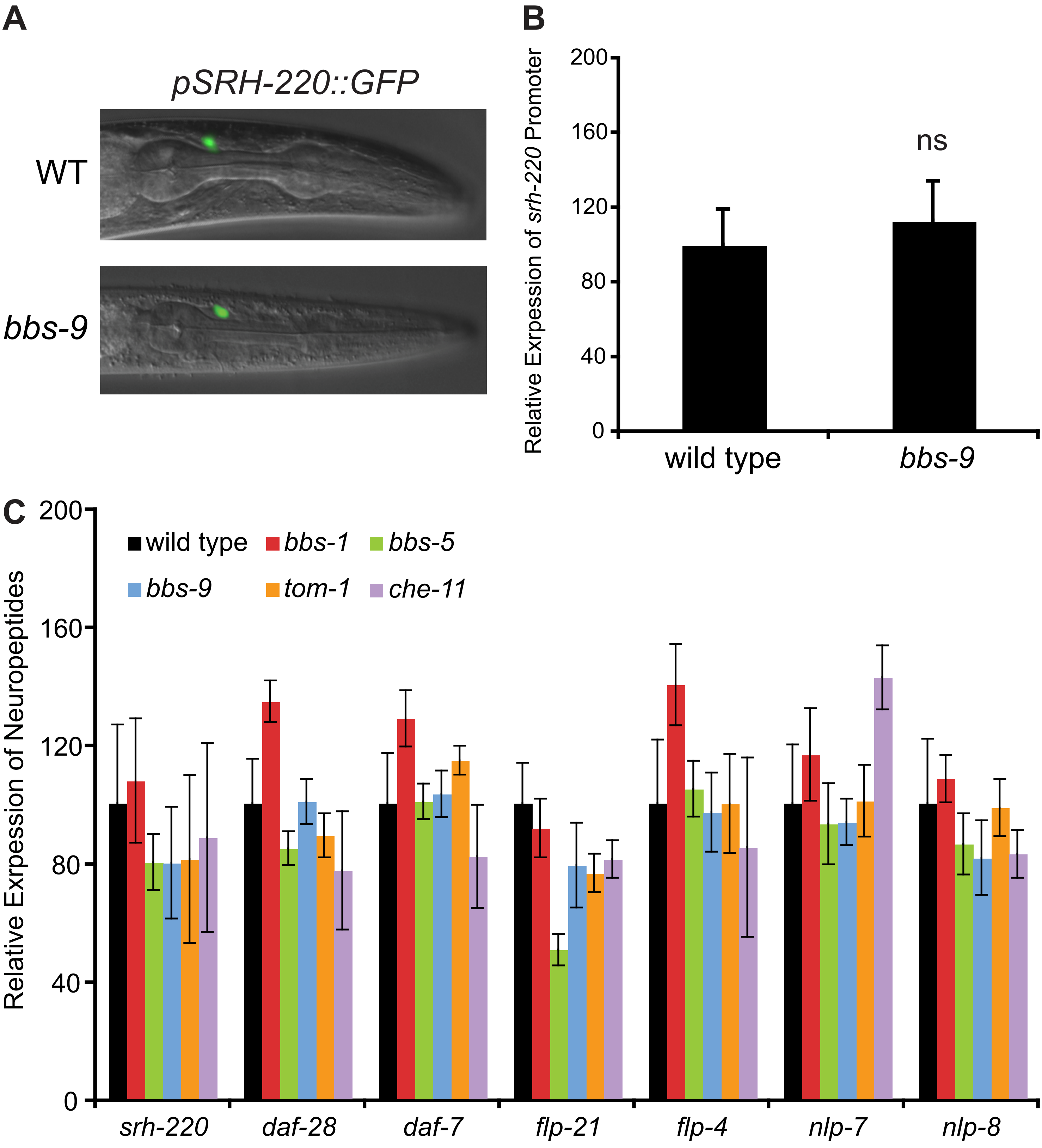

Supplemental Figure 3. BBsome Mutants have Normal Expression of Neuropeptide Genes

(A) Representative images of GFP expressed from the ADL specific *srh-220* promoter and (B) corresponding quantitations with standard error are shown. (ns = not significant). (C) Expression of various neuropeptide genes in BBSome, tomosyn and IFT mutants were quantified by RT-PCR. Expression was normalized to actin, *act-1.* Expression in the mutants were not statistically different from wild type except for *flp-21* in *bbs-5* mutant.
