# Supplementary material for: Hyperactive Neuroendocrine Secretion Causes Size, Feeding, and Metabolic Defects of C. elegans Bardet-Biedl Syndrome Mutants
Source: PLoS Biol. 2011 Dec 13;9(12):e1001219. doi: 10.1371/journal.pbio.1001219 (PMC3236739; doi:10.1371/journal.pbio.1001219)
Supplement: Figure S4 — Tomosyn and BBSome mutants have additive Nile Red phenotype. Pumping phenotype of BBSome mutants is not caused by increased expression of tph-1. (PDF) [file pbio.1001219.s004.pdf]

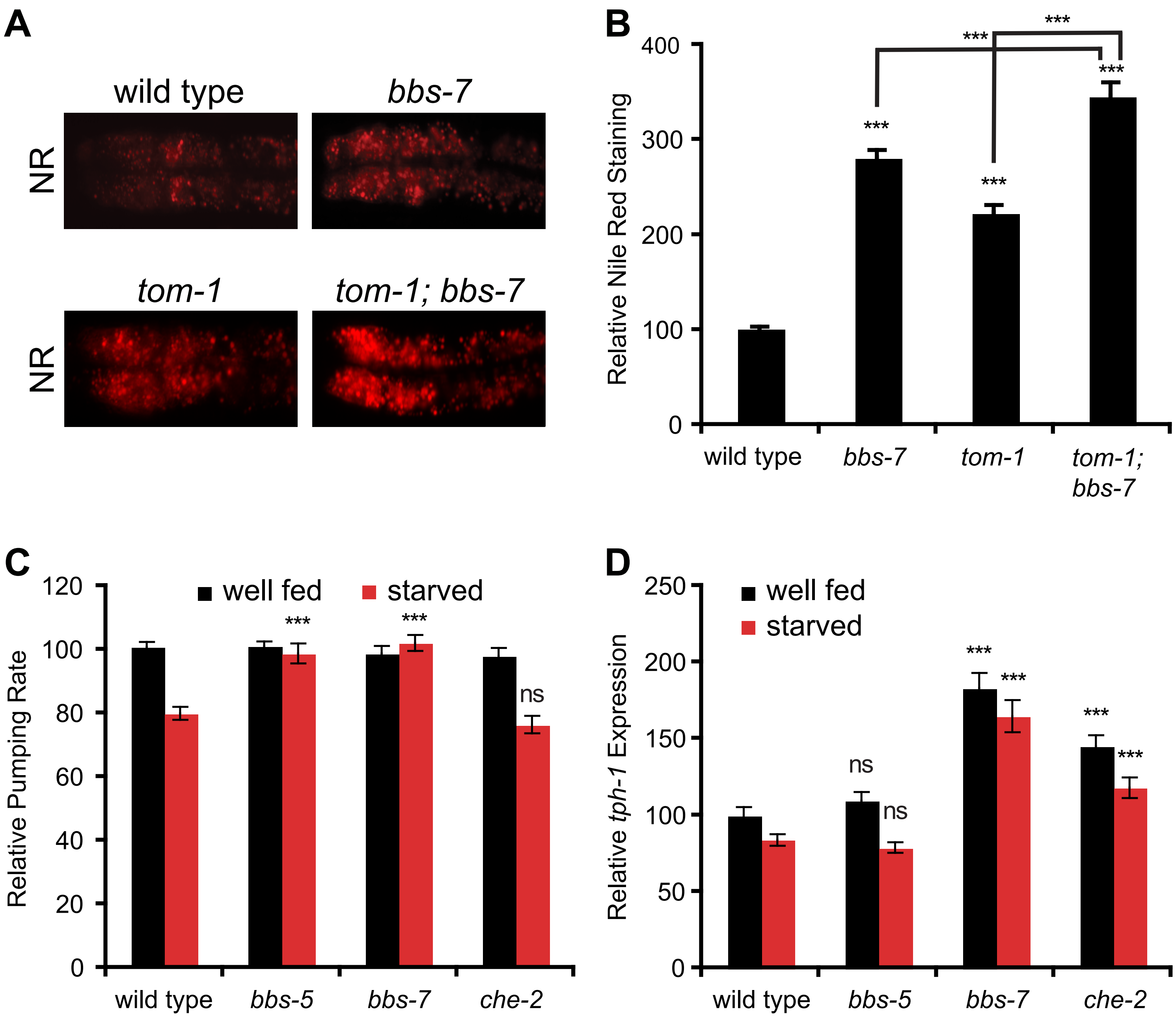

Supplemental Figure 4. Tomosyn and BBSome Mutants have Additive Nile Red Phenotype. Pumping Phenotype of BBSome Mutants is not Caused by Increased Expression of *tph-1*

Representative images (A) and quantitations with standard error (B) of Nile Red staining in the first pair of intestinal cells of *bbs-7* and *tom-1* mutants. (*** = p-value < 0.001 compared to wild type and comparing the double mutant to either single mutant).

Pumping rate (C) and *tph-1* expression in ADF neurons (D) of BBSome and IFT mutant, *che-2* under well fed (black) and upon 3hrs of starvation (red). (*** = p-value < 0.001, ns = not significant compared to the respective wild type). *bbs* mutants have elevated pumping rate upon fasting whereas *che-2* mutants have wild type pumping rate despite increased expression of *tph-1* in ADF neurons suggesting increased expression of *tph-1* and increase release of serotonin are separable phenotypes.
