# Supplementary material for: Hyperactive Neuroendocrine Secretion Causes Size, Feeding, and Metabolic Defects of C. elegans Bardet-Biedl Syndrome Mutants
Source: PLoS Biol. 2011 Dec 13;9(12):e1001219. doi: 10.1371/journal.pbio.1001219 (PMC3236739; doi:10.1371/journal.pbio.1001219)
Supplement: Figure S5 — Dense-core vesicle marker, IDA-1, localized to cilia in bbs mutants. (PDF) [file pbio.1001219.s005.pdf]

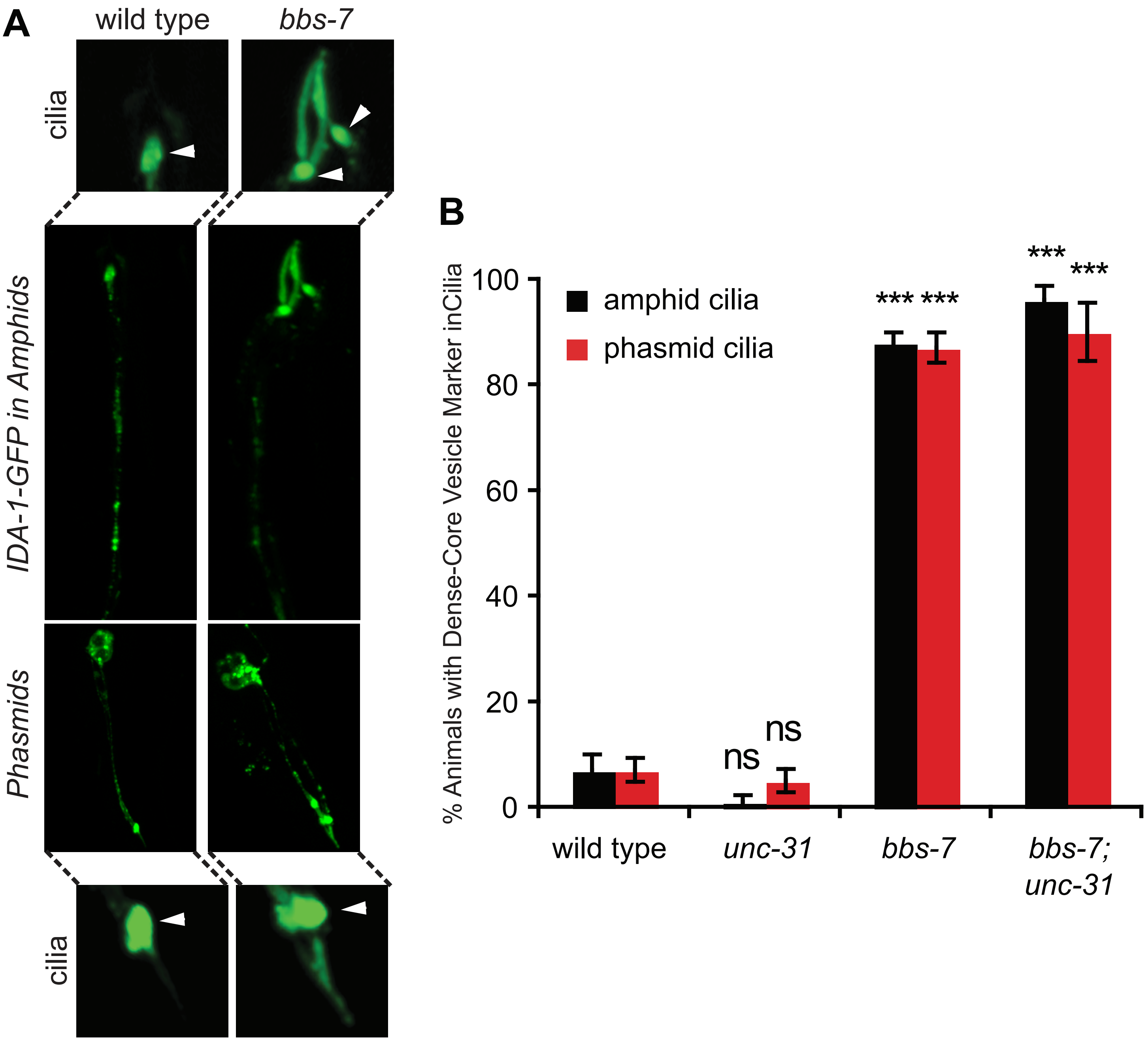

Supplemental Figure 5. Dense-Core Vesicle Marker, IDA-1, Localized to Cilia in *bbs* Mutants

Representative images (A) and quantitations with standard error (B) of IDA-1-GFP in amphid and phasmid cilia. Arrowhead indicate dendritic tip at the base of the cilia. (*** = p-value < 0.001, ns = not significant compare to wild type).
