# Supplementary material for: Hyperactive Neuroendocrine Secretion Causes Size, Feeding, and Metabolic Defects of C. elegans Bardet-Biedl Syndrome Mutants
Source: PLoS Biol. 2011 Dec 13;9(12):e1001219. doi: 10.1371/journal.pbio.1001219 (PMC3236739; doi:10.1371/journal.pbio.1001219)
Supplement: Table S1 — Tissue-specific rescue of bbs mutants. (PDF) [file pbio.1001219.s006.pdf]

**Supplemental Online Material**

**Supplemental Table 1. Tissue-Specific Rescue of *bbs* Mutants**

| Promoter | Promoter length [a] | Expression pattern | cDNA | Rescue Secretion [b] | Rescue Body Size [b] | Rescue Feeding [b] |
|---|---|---|---|---|---|---|
| *bbs-7* | 1452bp | Ciliated neurons | *bbs-1* *or* *bbs-7* | nd[c]<br>nd[c] | full<br>nd[c] | full<br>full |
| *bbs-1* | 2000bp | Ciliated neurons | *bbs-1* | full | nd[c] | nd[c] |
| *tax-4* | 2000bp | AWC, ASI, AFD, ASG, ASJ, ASK, BAG, URX, ASE | *bbs-1* *or* *bbs-7* | none<br>nd[c] | none<br>none | none<br>none |
| *ocr-2* | 2000bp | **ADL**, ASH, AWA, **ADF**, and phasmids | *bbs-1* *or* *bbs-7* | full<br>full | full<br>full | full<br>full |
| *gpa-13* | 2000bp | ASH, AWC, **ADF**, phasmids | *bbs-1* *or* *bbs-7* | nd[c]<br>nd[c] | partial<br>partial | full<br>full |
| *gpa-11* | 2000bp | **ADL,** ASH | *bbs-1* *or* *bbs-7* | nd[c]<br>nd[c] | partial<br>partial | nd[c]<br>nd[c] |
| *srh-220* | 1800bp | **ADL** | *bbs-1* *or* *bbs-7* | partial<br>partial | partial<br>partial | none<br>none |
| *tph-1* | 1300bp | **ADF** | *bbs-1* *or* *bbs-7* | nd[c]<br>nd[c] | none<br>none | full<br>full |
| *odr-7* | 1500bp | AWA | *bbs-1* *or* *bbs-7* | nd[c]<br>nd[c] | none<br>none | nd[c]<br>nd[c] |
| *hsp-16.1* | 422bp | all cells upon heat shock | *bbs-7* | partial | nd[c] | nd[c] |

Ciliated neurons that rescue *bbs* mutants are shown in bold.
[a] Promoter lengths are calculated from the start codon of the corresponding genes.
[b] Rescues were defined as reversion of the size, pumping rate when starved, and increased insulin secretion from ADL to wild type levels. Transgenic animals with intermediate phenotypes that were statistically different from both non-transgenic siblings and wild type were considered as partially rescued.
[c] not determined.
